# Impact of COVID-19 on Clinical Research Units (CRUs)

**DOI:** 10.1017/cts.2021.836

**Published:** 2021-08-13

**Authors:** Maran Subramain, Jackline M. Wangui-Verry, Kimberly J. Sprenger, Alejandro P. Comellas, Patrick B. Barlow

**Affiliations:** 1 Institute for Clinical and Translational Science, University of Iowa, Iowa City, IA, USA; 2 Department of Internal Medicine, University of Iowa, Iowa City, IA, USA

**Keywords:** COVID-19 pandemic, CRUs, CTSA CRUs, Clinical Research Units, Research Units Network

## Abstract

Few studies have explored the challenges that the COVID-19 pandemic has presented for Clinical Research Units (CRUs), the solutions that have been implemented, and the changes that have been made in the operational guidelines for these entities. This study sought to identify and document common practices implemented by CRUs around the United States of America (USA) when addressing the unique challenges posed by the COVID-19 pandemic. This descriptive study utilized a non-experimental mixed-methods approach and gathered data from representatives of 43 CRUs across the USA. An online survey was followed by in-depth interviews. The findings show that challenges faced from the COVID-19 pandemic, changes made to daily operations, and lessons learned are very similar across CRUs. Although most CRUs never stopped performing essential clinical research, many adapted to the pandemic by engaging in virtual visits, and many played key roles in administering and supporting both COVID-19 therapeutic and vaccine trials. Follow-up interviews showed that processes for formal approval and reopening were similar across CRUs. In addition to highlighting the significance of the role played by CRUs during the COVID-19 pandemic, this study addresses the relevance of CRUs and lays the groundwork for future conversations on the importance of these units.

## Introduction

In addition to driving significant changes in the personal and professional lives of individuals, the COVID-19 outbreak has posed critical challenges for the research and medical communities [1]. Hospitals, research institutes, and other academic institutions have been instrumental in the testing of COVID-19 vaccines while also continuing their groundbreaking human studies aimed at advancing medical science and improving clinical care.

The Clinical Research Units (CRUs), also known as Clinical Research Centers or Clinical and Translational Research Centers, were designed to provide both physical space and experienced interdisciplinary support staff to conduct and complete clinical research at hospitals, research institutes, and other academic institutions. By offering a one-stop shop for research support, CRUs consolidate institutional resources and encourage investigators to utilize the full range of support services. Many CRUs are also part of the Clinical and Translational Science Award (CTSA) program, thus furthering the CTSA’s mission in promoting research integration across the lifespan and catalyzing innovative clinical and translational research.

The Research Unit Network (RUN) is a national association of CRUs from both CTSA and non-CTSA institutions. Led by the University of Iowa CRU, RUN was created with the objective of enabling direct communication, sharing, and collaboration among CRUs. An intermediate goal of RUN is to identify the most successful practices among those that have been established and tested by individual CRUs, with the goal of sharing these practices for adoption by other CRUs.

The COVID-19 pandemic presented a natural experimental setting for RUN to study the impact of this pandemic on the functioning of CRUs. This study aimed to provide an overview of how CRUs were addressing COVID-19 and adapting to the emerging barriers, documenting practices implemented at participating institutions, and fostering data-driven decision-making in this unprecedented time.

## Methods

### Study Approval

The University of Iowa Institutional Review Board (IRB) reviewed this study, including the survey and invitation emails, and determined that it did not meet the federal definition of human subjects research; therefore, it did not require full review and approval by the IRB.

### Study Design

This study is a descriptive, mixed-methods, natural experiment, i.e., one that is conducted for the purpose of describing the characteristics of certain phenomena or selected variables without changing any aspect of the research setting (participants, treatments, or a dosage of treatment) [2]. In this study, quantitative data were gathered using an online survey and qualitative data were collected from in-depth follow-up interviews. Methodological pluralism was used for this study to gather richer data and to minimize the limitations that might have been associated with the mono method or a single data collection approach [3].

### Participants

All 43 CRUs in RUN (eTable 1 in the Supplement) were eligible to participate in the study because they were all impacted by the COVID-19 pandemic. Representatives of the CRUs included principal investigators (PIs), unit directors, and nurses. All are key personnel who lead the day-to-day administration and operations of CRUs at their institutes. Additionally, a purposeful sampling approach was used when selecting the interview respondents of this study. Purposeful sampling is a research technique that identifies and selects individuals who are experts and well versed with an occurrence of interest, and willing to discuss their knowledge and experience in an expressive and reflective way [4].

### Data Collection

Representatives from all 43 RUN member institutes were invited to complete an online survey that was comprised of 28 questions about the impact of COVID-19. The web-based survey was administered using the Qualtrics software, between April 21, 2020 and May 8, 2020, as the United States of America (USA) saw its first spike in COVID-19 cases. Semi-structured follow-up interviews were conducted with representatives of CRUs at seven targeted and geographically representative RUN members (Southeast, Southwest, Midwest, Northeast, South Central regions) to document their insights on topics such as processes for returning to research operations, new COVID-19 therapeutic and vaccine trials administered, and lessons learned. All interviews were conducted in August 2020.

To add to the context of our study results, we have used publicly available data from the COVID-19 Atlas [5] to calculate the impact of the COVID-19 pandemic on the counties in which RUN member institutions are located both during the initial member survey and over the time of the follow-up interviews. For each county, the mean rolling 7-day average number of new confirmed cases per day was averaged for the data collection period, divided by the county total population, and then multiplied by 100,000 to produce the mean 7-day rolling average number of new cases for each county per 100,000 population.

### Data Analysis

Descriptive data analyses were used to organize the information gathered via the online survey. Open and axial coding were used to identify general categories of information obtained in the interviews, and to sort those categories into related and meaningful groups, respectively. The qualitative data were complementary to the quantitative information gathered. Moreover, in-depth details of the qualitative data confirm, explain, and relate to the quantitative information observed in this study.

## Results

**Figure 1.**
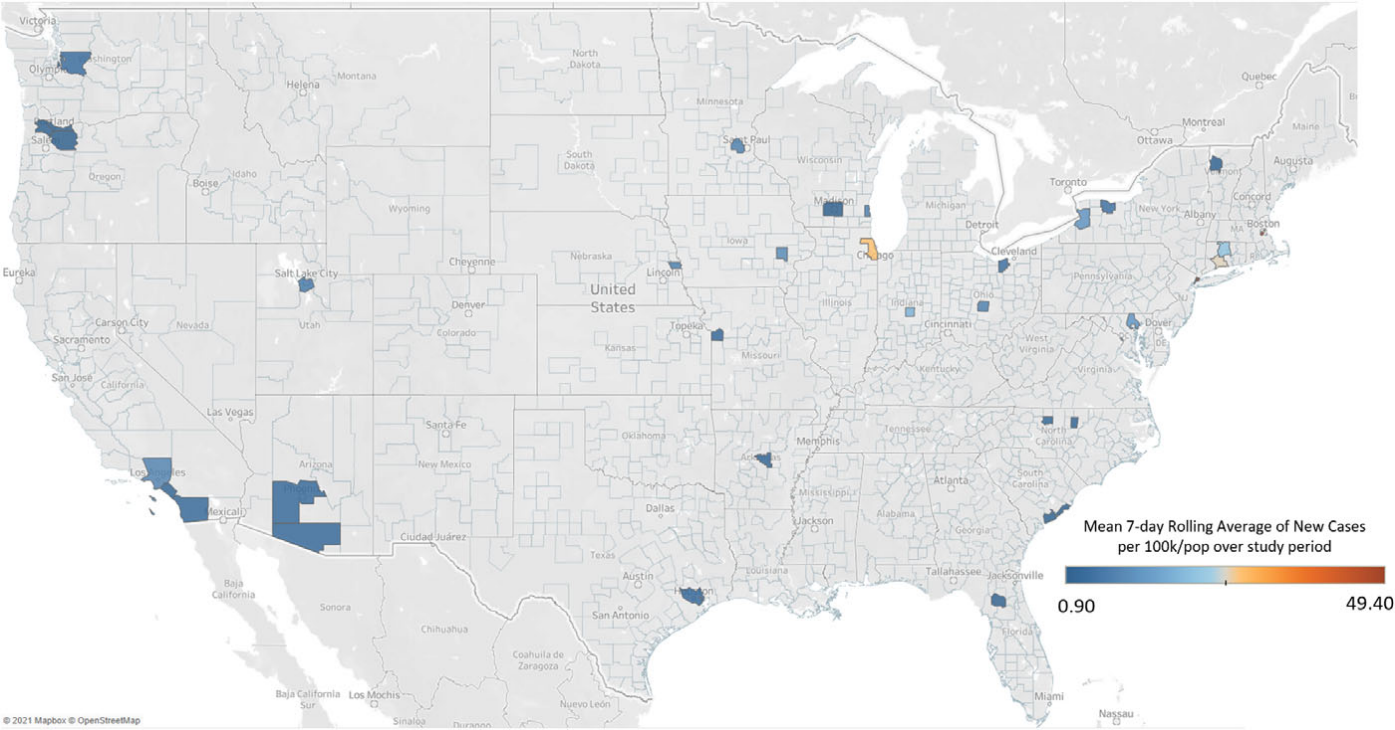
Mean 7-day rolling average of new cases per 100k/population for Research Unit Network (RUN) member counties during survey period of April 21, 2020–May 8, 2020.

**Figure 2.**
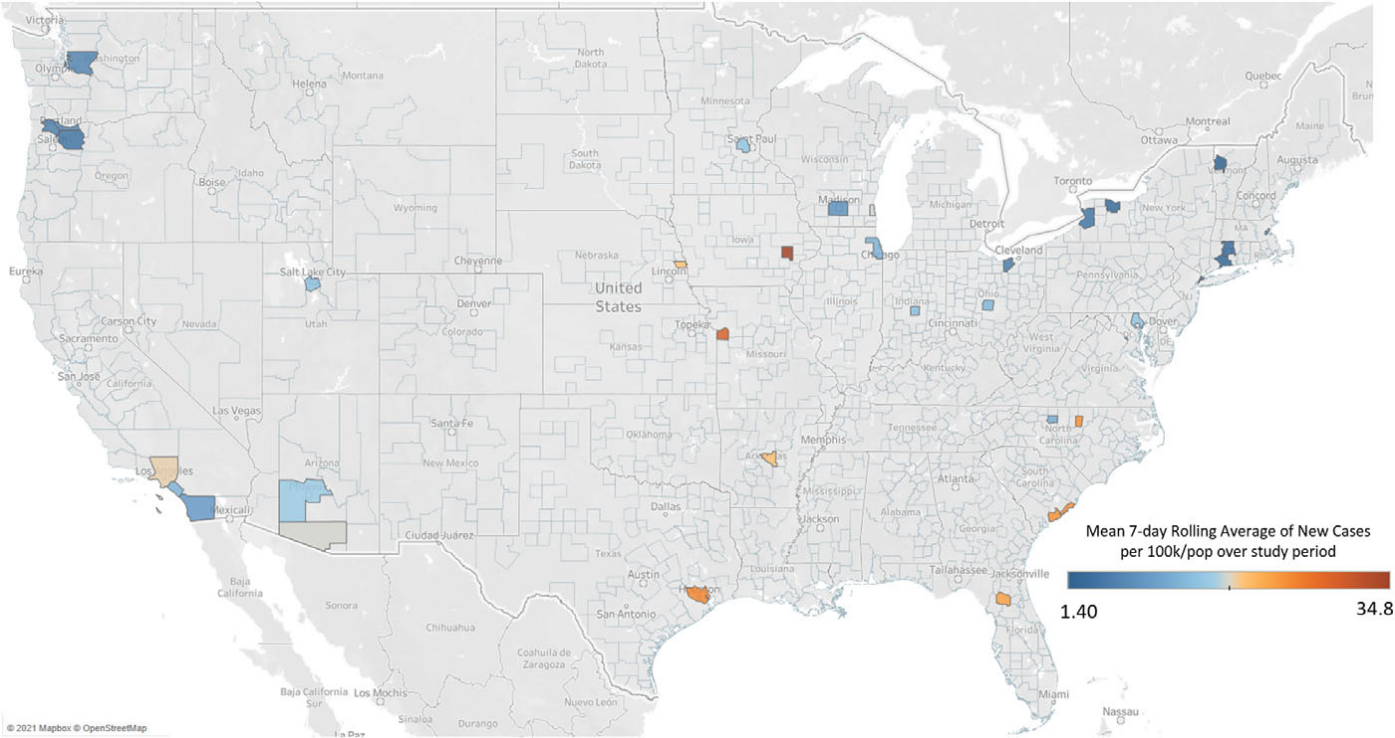
Mean 7-day rolling average of new cases per 100k/population for RUN member counties during interview period of August 1, 2020–August 31, 2020.

Figures 1 and 2 show the mean 7-day rolling average number of new cases per 100,000 population for each county in which a RUN member institution is located during the survey period (Fig. 1) and interview period (Fig. 2). All RUN members were included in these descriptive maps to protect the privacy of study participants. The survey was conducted during the first wave of COVID-19 infections, which primarily hit RUN member institutions in New York City, Boston, and, to a lesser extent, Chicago. Comparatively, the interviews during August happened during the second wave where the Midwest, Southeast, and Southwest were far more impacted.

### Description of Sample

The initial email survey invitations were sent to 43 individual RUN institutions. Twenty-nine total survey responses (67%) were included in the final analysis. Seven of the eight targeted survey respondents also participated in follow-up interviews. No contradicting information was obtained from quantitative versus qualitative data. Furthermore, 90% (26 of 29) of survey respondents were from institutes that received CTSA funding in 2020, as were 100% of interview respondents. Table 1 provides additional detail on comparing the responders and nonresponders to the survey.

**Table 1.** Nonresponder analysis for RUN survey

Comparison variable	Responded to survey frequency (%)	
	Yes (*n* = 29)	No (*n* = 14)
*Geographic region*		
West	5 (17.2%)	5 (35.7%)
Southwest	1 (3.4%)	2 (14.3%)
Midwest	11 (37.9%)	0 (0%)
Southeast	4 (13.8%)	2 (14.3%)
Northeast	8 (27.6%)	5 (35.7%)
*Type of institution*		
Public	21 (72.4%)	11 (78.6%)
Private	8 (27.6%)	3 (21.4%)
*CTSA funding*		
Yes	26 (89.7%)	10 (71.4%)
No	3 (10.3%)	4 (28.6%)

RUN, Research Unit Network; CTSA, Clinical and Translational Science Award.

### Descriptive Analysis

Table 2 details the actions taken by both institutions and research sponsors in response to the pandemic. Although the COVID-19 pandemic impacted all CRUs, during the period in which the survey was administered (April 2020), 79% (*n* = 23) continued to perform essential clinical research (that which cannot be performed remotely and is essential to a participant’s health and/or well-being) [6]. Also notable is that 86% of institutions (25 of 29) had stopped all nonessential research (that which cannot be performed remotely and is nonessential to a participant’s health and/or well-being) during this time.

**Table 2.** Institutional and research sponsor reaction to initial COVID-19 shutdown

Item text, response options	Frequency (%)
*How has the COVID-19 pandemic impacted research activities at your unit?*	
Stopping of all nonessential research	25 (86.2%)
Continuing to provide essential clinical research	23 (79.3%)
Reduced hours of operations for essential research	12 (41.4%)
Stopped all research	1 (3.4%)
There have been no changes to research activities	0 (0%)
*How have sponsors influenced your research activities?*	
Paused enrollment	27 (93.1%)
Virtual visits	23 (79.3%)
Paused visits	19 (65.5%)
Closed studies	7 (24.1%)
Other	3 (10.3%)
No changes have occurred	1 (3.4%)

Participants could choose multiple response options for both items.

Although 93% (*n* = 27) of CRUs paused the enrollment of participants in certain studies, many CRUs (79%, or *n* = 23) continued to engage in virtual research visits. These data further support the notion that the COVID-19 pandemic has led to the expansion of not only the practice of telemedicine [7], but also virtual research. Finally, more than 17% (*n* = 5) of CRUs were seeing both COVID-19 and non-COVID-19 research participants in their units at the time of the survey, and 80% (*n* = 24) were participating in at least one COVID-19 therapeutic or vaccine-related trial.

Table 3 breaks down specific changes CRUs reported making to their standard procedures during the first months of the pandemic. For the question on the usage of personal protective equipment (PPE) in CRUs during this pandemic, the most commonly listed items were normal surgical masks (100%, *n* = 29), gloves (86%, *n* = 25), gowns (76%, *n* = 22), and face shields (59.6%, *n* = 17). Hospitals (83%, *n* = 24) and CRUs (52%, *n* = 15) provided and/or funded PPEs. Additionally, PPE needs were supplemented by donations gathered by the University Employee Health and Clinical and Translational Science Institutes.

**Table 3.** Changes to Clinical Research Unit (CRU) standards of procedure and staffing due to COVID-19

Item text, response options	Frequency (%)
*Are you currently seeing BOTH COVID-19 and non-COVID-19 subjects in your research units? (n = 28)*	
Yes	5 (17.9%)
No	23 (82.1%)
What PPE is being used by your site for interactions your research subjects?*	
Normal surgical masks	29 (100%)
Gloves	25 (86.2%)
Gowns	22 (75.9%)
Face shields	17 (58.6%)
Goggles	12 (41.4%)
N95 respirators	12 (41.4%)
Other types of masks (please specify, e.g. cloth masks)	8 (27.6%)
Head covering	5 (17.2%)
Shoe covers	4 (13.8%)
CAPR/PAPR	1 (3.4%)
*Have you developed or asked to make specific changes in guidelines for care of research patients who require PPE for non-COVID-19 reasons? (e.g., do not use PPE for MDRO to conserve PPE) (n = 28):*	
Yes	8 (28.6%)
No	20 (71.4%)
Who is providing/funding PPE?*	
Hospital	24 (82.8%)
CRU providing	15 (51.7%)
Research teams	7 (24.1%)
CRU staff/self-acquired	4 (13.8%)
Others	4 (13.8%)
Sponsor	4 (13.8%)
Is your CRU currently being used for other purposes?*	
No	23 (79.3%)
Yes, for non-COVID-19 patients	3 (10.3%)
Yes, for some other purpose (please specify)	3 (10.3%)
Yes, for COVID-19 patients	2 (6.9%)
Has your any of your CRU Staff been reassigned as a result of COVID-19?*	
No	18 (62.1%)
Yes, voluntary reassignment	10 (34.5%)
Yes, mandatory reassignment	3 (10.3%)
*Which are being reassigned? (n = 13)*	
Nurses	10 (76.9%)
Clerks/secretary	5 (38.5%)
Other	4 (30.8%)
Dietician	2 (15.4%)
Medical assistants	2 (15.4%)
Nurse assistants	2 (15.4%)
Lab staff	1 (7.7%)
Respiratory therapist	1 (7.7%)
*Q 13. Do you currently have any CRU staff in quarantine due to COVID-19 exposure?*	
Yes	2 (6.9%)
No	27 (93.1%)

PPE, personal protective equipment; CAPR, controlled air-purifying respirator; PAPR, powered air-purifying respirators; MDRO, multidrug-resistant organisms; CRU, Clinical Research Unit.

* Participants could select multiple response options.

At the beginning of the pandemic, 16 CRUs (57%) devoted more than the usual time developing and revising standard operating procedures (SOPs) and organization of their labs/units. Additionally, 14 CRUs (50%) spent more than the usual time on education and professional development as their usual operations slowed down.

Table 4 outlines how respondents reported their communication practices both within their unit and externally to their study teams. CRUs communicated with research teams consistently but at different frequencies, with 52% (*n* = 15) weekly, 17% (*n* = 5) daily, and 21% (*n* = 6) as needed. The most common methods of communication were email (97%, *n* = 28), phone (45%, *n* = 13), and video conference (45%, *n* = 13). Also, more than 60% (*n* = 17) of respondents indicated they had already met or have planned an initial meeting to develop a recovery plan as a strategy to return to normal operations.

**Table 4.** Communications and planning strategies used by CRUs

Item text, response options	Frequency (%)
*Are you maintaining your regular communications with your research teams?*	
Yes	28 (96.5%)
No	1 (3.5%)
*How frequently have you been providing regular communications with your research teams?*	
Weekly	15 (51.7%)
Other e.g., “as needed”	6 (20.7%)
Daily	5 (17.2%)
Every 2 weeks	1 (3.4%)
Monthly	1 (3.4%)
What format(s) are these communications?*	
Email	28 (96.6%)
Phone	13 (44.8%)
Video Conference (e.g., Zoom)	13 (44.8%)
Newsletter	4 (13.8%)
Other	2 (6.9%)
*Has your CRU staff began meeting to develop a plan and/or timeline for recovery (i.e., returning to normal operations)?*	
Yes, we are already meeting	15 (51.7%)
No, and we have not planned an initial meeting	11 (37.9%)
No, but we have planned an initial meeting	3 (10.3%)

CRU, Clinical Research Unit.

* Participants could select multiple response options.

As previously noted, 80% of the survey respondents indicated that their unit was involved with one-or-more COVID-19 therapeutic or vaccine-related trials. Table 5 provides a summary of the trials that were listed. We used data from www.clinicaltrials.gov to match any impartial information and study identification numbers to the proper clinical trial name using (1) incomplete trial title, (2) drug name, (3) sponsor name, and (4) institution name as necessary. Any response that did not provide a conclusive result using those parameters is listed under “Unspecified Trial Name.”

**Table 5.** Specific COVID-19 clinical trials and other studies reported by participants

Specific trial or study name	Unspecified study responses
A multicenter, adaptive, randomized blinded controlled trial of the safety and efficacy of investigational therapeutics for the treatment of COVID-19 in hospitalized adults, 20-0006.	Collection study
A Phase 2 multiple dose study to evaluate the efficacy and safety of PUL-042 inhalation solution in reducing the infection rate and progression to COVID-19 in adults exposed to SARS-CoV-2	Biorepository – local MD
A Phase 2 multiple dose study to evaluate the efficacy and safety of PUL-042 inhalation solution in reducing the severity of COVID-19 in adults positive for SARS-CoV-2 infection	Convalescent plasma
A Phase 2, open-label, randomized, multicenter study to investigatge the pharmacodynamics, pharmacokinetics, safety, and efficacy of 8 mg/kg or 4 mg/kg intravenous tociluzumab in patients with moderate-to-severe COVID-19 pneumonia	Convalescent plasma donor protocol – outpatient
Adaptive COVID-19 treatment trial (ACTT)	Covered – COVID-19 evaluation of risk for emergency departments
Healthcare worker exposure response and outcomes (HERO) registry study	COVID-19 blood, sputum, swab, and urine study
Healthcare worker exposure response and outcomes of hydroxychloroquine (HERO-HCQ)	Emerging respiratory infections
Hydroxychloroquine for COVID-19 post-exposure prophylaxis (PEP)	Gimsilumab
Longitudinal assessment of serological status with respect to SARS-COV-2 and subsequent incidence of clinical COVID-19 disease in the healthcare workforce	Healthcare worker surveillance study
Hydroxychloroquine monotherapy and in combination with azithromycin in patients with moderate and severe COVID-19 disease	Hydroxychloroquine
Pediatric COVID-19 US registry	Inpatient viral swab replacement project
Randomized controlled trial of losartan for outpatients with COVID-19	Investigator initiated
Rapid in-home SARS-Co-V-2 IgG antibody testing to assess community-level immunity in healthcare workers working in high-risk exposure settings	IVY
RCT of losartan for outpatients with COVID-19	Mavrilimumab
RLF-100 for the prevention and treatment of acute respiratory distress syndrome in COVID-19 infection	PCORI
Study of efficacy and safety of canakinumab treatment for CRS in participants with COVID-19-induced pneumonia	Progesterone
Study of TJ003234 (anti-GM-CSF monoclonal antibody) in subjects with severe coronavirus disease 2019 (COVID-19)	PROTECT
Study to evaluate the efficacy and safety of leronlimab for mild-to-moderate COVID-19	Remdesivir Phases 1 and 2
Study to evaluate the efficacy and safety of leronlimab for patients with severe or critical coronavirus disease 2019 (COVID-19)	Selinexor
Study to evaluate the safety and antiviral activity of remdesivir (GS-5734™) in participants with moderate coronavirus disease (COVID-19) compared to standard of care treatment	Tocilizumab
Study to evaluate the safety and antiviral activity of remdesivir (GS-5734™) in participants with severe coronavirus disease (COVID-19)	Universal protocol
	Vaccine trial

Whenever possible, responses were matched to the full study name by the provided the Study ID using www.ClinicalTrials.Gov. Partial responses were matched to the full study name using drug name(s), study abbreviation, institution, and time of the survey. Finally, responses that could not be reliably connected to a study using one of those other methods were classified as “Non-specific Responses.”

### Observations from Follow-up Interviews and Key Themes

Semi-structured follow-up interviews were conducted with CRU leaders (directors, assistant directors, nurse managers) at seven targeted and geographically representative RUN institutes. General categories of information contained in interview transcripts were identified using open coding. Then, tentative labels were organized into related and meaningful groups of data using axial coding, which further assisted authors in refining, aligning, and categorizing the data into the distinct themes [8] described in the following sections. A summary of the key changes CRUs reported making as well as how many of our interview participants (*n* = 7) reported each change can be found in Table 6.

**Table 6.** Changes made to CRU operating procedures due to COVID-19

	Changes to practice	Frequency (*n* = 7)
1.	Study teams were to contact participants within 24 hours prior to the visit for any COVID-19 symptoms and to explain the screening procedures during their visit.	6
2.	Limiting participant number allowed in the CRU at the same time	6
3.	Continue studies that are essential to a participant’s health and/or wellbeing or coincident with an in-house clinical care	4
4.	PIs had to provide plans explaining how they would conduct the studies safely given the pandemic restrictions.	4
5.	Subjects and staff following PPE guidelines	4
6.	Mandatory use of vacation time by CRU staff	2
7.	Conducting procedures outside the facility (e.g., Spirometry)	1
8.	Arranging for mail-order medications or locally-provided lab work (avoiding travel and exposure)	1
9.	Dividing research spaces into “clean” and “dirty” areas	1
10.	Designating special areas for immune-compromised individuals to protect them	1
11.	Providing special pods for COVID-19-positive participants	1

CRU, Clinical Research Unit; PI, principal investigator; PPE, personal protective equipment.

**Continuing essential research:** The CRUs representatives who were interviewed defined essential research similarly – as “research that is essential to a participant’s health and/or well-being.” These respondents reiterated that their CRUs never completely stopped research activities. These CRUs typically functioned at one-third to half capacity, and stopped all nonessential research, but remained continuously engaged in research activities to some extent, especially essential research. However, CRUs did elect to pause certain essential studies that involved high-risk participants (older participants, and also participants of any age with serious health problems such as cardiopulmonary conditions, immunosuppression, morbid obesity, or diabetes) [9].

**Approval process for restarting research activities:** Some institutes started reopening in stages. For example, Stage 1 focused on continuing studies or initiating studies that had prior approval. New protocols were not accepted at this first stage. During Stage 2, CRUs did review protocols and started those new protocols involving non-essential health care activities and study visits coincident with clinical care were allowed. During Stage 3, research could resume in university buildings and at off campus locations and facilities (schools, nursing homes, etc.) [10]. In general, studies were likely to be approved if they were in the same stage as indicated by the office of human subjects research – entities that provide leadership on operations and regulatory oversight of human research activities [11] in their respective institutes. When clinics began opening, CRUs slowly eased into the clinics with interventional studies where the participants were to be assigned to research (or health service) conditions.

The revision of SOPs to include COVID-19-related requirements and study prioritization (Essential, Interventional, Observational – in that order) was also similar across CRUs interviewed. Almost all CRU leaders (directors, assistant directors, nurse managers) were intimately involved in reopening discussions. Most institutes followed a top-down approach when approving the restart plans for individual studies. Generally, approval from the Vice President for Research (VPR), IRB, equivalent institutional leadership unit, or some combination of representatives of these groups was needed. Regardless of who ultimately approved the restart, PIs had to provide plans explaining how they would conduct their study safely given the pandemic restrictions. Before the CRUs reopened, each of them communicated with study teams and shared their SOPs so that they could make the necessary adjustments to the procedures for participant’s visits to the CRU.

**Participant safety, trust, and involvement:** Planning for the restoration of operations often involved meetings of CRU representatives with counterparts from the hospital Epidemiology, Environmental Health & Safety unit, or its equivalent. The latter provided guidelines for how the CRU could restart its research activities, including planning on involving COVID-19-positive research participants. Critical Research Units interviewed indicated that they have been following similar guiding principles and practices for study visits that were to be conducted in person. For example, study teams were to contact participants within 24 hours prior to a visit to confirm that the participant did not have any COVID-19 symptoms and to explain the screening procedures during their visit.

The willingness of individuals to participate in various clinical research studies attests to their trust in safety campaigns and safety procedures at medical and research institutes. In many cases, clinical research teams also have known the participants for several years and have gained their understanding and respect. Participants were more than willing to cooperate. Similar sentiments were described in a study published by Padala et al. [12], where 40 of 51 active research participants felt that “the medical center was well prepared and expressed that the additional screening put them at ease” during this pandemic.

**Adaptability:** The interviews revealed that CRUs made notable and creative adaptations in responding to the COVID-19 pandemic. Mainly, they adjusted procedures and operations. In terms of procedures, one CRU indicated that it conducted spirometry studies outside the facility. Participants were on the premises initially, but moved outside in the shade to complete the research visit. These outside locations had great appeal for younger participants and children. Other adaptations to procedures were made for research participants who were unable to come to the unit but needed treatment. In such cases, the CRU implemented a procedure for mailing drugs or arranged for lab work to be done locally so that participants could continue the study without interruptions. Some study sponsors were willing to pay for the costs associated with these changes.

An example of an effective operations change instituted by a CRU is the splitting of research areas into two, with one identified as “clean” (for participants who were not COVID-19 positive) and the other as “dirty” (for participants who were COVID-19 positive), for the completion of research visits. This resulted in the use of different hospital gowns and other PPE. Additionally, in some CRUs specific hallways were designated for use by immune-compromised individuals, and many CRUs limited the number of participants allowed in the unit. Other operational changes included purchasing patient/nurse pods for COVID-19-positive participants and setting them up in parking lots.

### COVID-19 Therapeutic and Vaccine Trials

As observed in both survey and interviews, CRUs have been involved in numerous COVID-19-related studies and vaccine trials throughout the pandemic. One interview respondent indicated that “it is almost a norm for CRUs to have at least one COVID study brought into the unit on a daily basis.” Notably, the CRUs have been involved in COVID-19 studies at different stages. Some therapeutic studies were conducted at the pre-exposure stage; others involved COVID-19 drugs and treatments for participants who tested positive; and others involved various vaccine trials. The most common pre-exposure trial engaged in by CRUs at the time of the interviews was the HERO-HCQ trial, which explored the effectiveness of hydroxychloroquine to prevent transmission of SARS-CoV-2 among healthcare workers. In addition, CRUs were involved in testing the effectiveness of drugs such as Remdesivir and Losartan in preventing the progression of symptoms in participants who were SARS-CoV-2 positive. Convalescent plasma and anti-spike monoclonal antibodies were among other studies actively pursued by CRUs in patients admitted to the hospital. Clinical Research Units have also been actively involved in vaccine trials including Moderna, Pfizer, and AstraZeneca.

### Challenges Faced and Key to Success

No major challenges related to university or hospital leadership or bureaucracy were brought up by interview participants. However, CRU managers dealt with a variety of difficult staffing issues during the pandemic. For example, the need for staff at some institutions to use vacation time disrupted daily CRU operations.

CRU success was found to be critically dependent on supportive and engaged leadership. Study respondents cited the following as key themes related to the responses of leaders who had proven effective: adapting to rapid changes, focusing on mental health/resilience of staff members, being optimistic, and keeping up staff morale. Similar approaches have been noted in other studies that explore leadership traits and their important role during this pandemic [13,14]. Shingler-Nace [14] identified five distinct elements to successful leadership during the COVID-19 crisis, which were staying calm, communicating, collaborating, coordinating, and providing support.

The CRU representatives who were interviewed reported striving to stay up to date with infection control and/or CDC requirements. They also reported that grant PIs are generally very involved in making sure everyone is up to date on changes to the research enterprise. Similar themes were noted in an article published by the Clinical and Translational Science Collaborative of Case Western Reserve University; adaptability to new challenges was defined as vital for clinical research professionals during this pandemic [15].

## Discussion

Currently, there is a renewed awareness of the importance of virtual care, proactive investment in public health infrastructure, and the preparedness for a robust public health system that can effectively respond to epidemics like the COVID-19 pandemic [16,17]. The first CRU was established in 1910 when the first American hospital entirely devoted to clinical research was created at Rockefeller University. However, in 2014, the National Center for Advancing Translational Sciences (NCATS) of the National Institutes of Health (NIH) announced that direct support of the CRUs would no longer be allowed for any CTSA, leading to the need for hospitals and other research institutes to operate these entities on a service center model [18,19]. Despite this loss of direct CTSA support for CRUs, the contributions of these units to the research and medical communities have been irreplaceable, especially during the COVID-19 pandemic.

This study provides evidence for the critical role played by CRUs during the COVID-19 pandemic, lessons learned, adaptability, and critical components for CRU success. It demonstrates that CRUs never stopped essential clinical research, and that a high percentage of CRUs adapted to the pandemic by engaging in virtual visits. Most importantly, CRUs played key roles in administering and supporting COVID-19 and vaccine trials, including their significant contribution in Phase 3 trials for the Pfizer and Moderna vaccines, the first two vaccines approved for use in the USA. Additionally, flexibility, willingness to adapt to constant changes, supportive and engaged leadership were identified as the critical components for success of these CRUs in the extraordinary pandemic environment.

The identification of common practices of CRUs by this mixed-methods study is expected to inform CRU operations in the future, and hopefully will draw the attention of NCATS and other entities to the need for proper support for these units, as well as to recognize the vital contributions CRUs make to broadly define medical research and progress across all medical fields.

## Limitations

This study has several limitations which include the modest sample size for gathering sufficient data that is representative and generalizable to all CRUs across the USA. The collection and integration of other longitudinal and additional relevant data from current and new RUN members on a continuous basis is expected to provide richer perspectives and a more holistic view of the contributions CRUs are making to COVID-19 studies, and to clinical studies in general.

## Supplementary material

For supplementary material accompanying this paper visit http://dx.doi.org/10.1017/cts.2021.836. click here to view supplementary material
