# Supplementary material for: Impact of COVID-19 on Clinical Research Units (CRUs)
Source: J Clin Transl Sci. 2021 Aug 13;5(1):e167. doi: 10.1017/cts.2021.836 (PMC8503072; doi:10.1017/cts.2021.836)
Supplement: Supplementary file 1 [file S2059866121008360sup001.docx]

Supplementary Online Content

eTable 1. Research Unit Network (RUN) Members Institutes (as of April 2020)

eFigure 1. Impact of Covid-19 On CRUs – RUN Members Online Survey Questions

eFigure 2. Impact of Covid-19 On CRUs – RUN Members Interview Guide

**eTable 1. Research Unit Network (RUN) Members Institutes (as of April 2020)**

- Arizona State University
- Boston Children’s Hospital (Harvard)
- Cedars-Sinai Hospital – Los Angeles, CA
- Charles R. Drew University of Medicine and Science – Los Angeles, CA
- Cleveland Clinic
- Columbia University
- Einstein and Montefiore
- Georgetown University Medical Center
- Indiana University
- Johns Hopkins All Children’s Hospital
- Massachusetts General Hospital
- Medical College of Wisconsin
- Medical University of South Carolina
- New York Presbyterian Hospital Weill Cornell Medical Center
- Northwestern University Clinical and Translational Sciences
- NYU Langone Health
- Oregon Health and Science University
- The Ohio State University
- The University of Vermont Medical Center
- University of Arizona
- University of Arkansas for Medical Sciences
- University of Buffalo
- University of California, Irvine
- University of California, Los Angeles
- University of California, Lundquist Institute, Torrance
- University of California, San Diego
- University of California, San Francisco
- University of Connecticut / UConn Health
- University of Florida
- University of Illinois, Chicago
- University of Iowa
- University of Kansas
- University of Maryland
- University of Minnesota
- University of Nebraska
- University of North Carolina at Chapel Hill
- University of Rochester Medical Center
- University of Texas Health Science Center at Houston
- University of Utah Health
- University of Washington at Seattle
- University of Wisconsin, Madison
- Wake Forest University
- Yale Center for Clinical Investigations / YNHH

eFigure 1. Impact of Covid-19 On CRUs – RUN Members Online Survey Questions

Q1. Research Unit Network (RUN) Survey: Impact of COVID-19 on Clinical Research Units The purpose of this survey is to assess how Clinical Research Units are addressing and adapting to emerging COVID-19 pandemic barriers. The deadline to respond is 5:00 PM ET on Tuesday, April 28, 2020. We appreciate your timely response and will be sending reminder emails.

Q2. How has the COVID-19 pandemic impacted research activities at your Unit? (check all that apply)

- There have been no changes to research activities
- Stopped all research
- Stopping of all non-essential research
- Reduced hours of operations for essential research
- Continuing to provide essential clinical research

Q3. How have sponsors influenced your research activities? (check all that apply)

- No changes have occurred
- Paused enrollment
- Paused visits
- Virtual visits
- Closed studies
- Other (please specify): ________________________________________________

Q4. Were you provided a definition of “Essential Research”? If so, please provide below. We would prefer if you could copy and paste the exact definition you were given instead of paraphrasing it.

________________________________________________________________

________________________________________________________________

________________________________________________________________

________________________________________________________________

________________________________________________________________

Q5. You may also upload a copy of the definition, such as a PDF of an email or other document, if you prefer.

Q6. Is your CRU involved with one or more COVID-19 trials?

- Yes
- No

Q7. Name(s) of the COVID-19 trial(s):

|  | Trial Name | Trial Number (if available) |
| --- | --- | --- |
| Trial One |  |  |
| Trial Two |  |  |
| Trial Three |  |  |
| Trial Four |  |  |
| Trial Five |  |  |

Q8. Please list your additional COVID-19 Trials' Names and Numbers here using one line per trial.

________________________________________________________________

________________________________________________________________

________________________________________________________________

________________________________________________________________

________________________________________________________________

Q9. Are you currently seeing **BOTH** COVID-19 and non-COVID-19 subjects in your research units?

- Yes
- No

Q10. How are you accommodating both subjects in your research units?

________________________________________________________________

________________________________________________________________

________________________________________________________________

________________________________________________________________

________________________________________________________________

Q11. Again, if you prefer, you may upload a document describing your accommodations if you have it.

Q12. What PPE is being used by your site for interactions your research subjects? (check all that apply)

- N95 respirators
- Normal surgical masks
- Other types of masks (please specify, e.g. cloth masks) ________________________________________________
- Goggles
- Face shields
- Gloves
- Gowns
- Head covering
- Shoe covers
- CAPR/PAPR

Q13. Have you developed or asked to make specific changes in guidelines for care of research patients who require PPE for non-COVID-19 reasons? (e.g., do not use PPE for MDRO to conserve PPE):

- Yes
- No

Q14. Please describe these changes.

________________________________________________________________

________________________________________________________________

________________________________________________________________

________________________________________________________________

________________________________________________________________

Q15. Who is providing/funding PPE? (check all that apply)

- Sponsor
- Hospital
- Research teams
- CRU staff/self-acquired
- CRU providing
- Other (please provide additional comments you would like to share) ________________________________________________

Q16. Is your CRU currently being used for other purposes? (check all that apply)

- No
- Yes, for COVID-19 patients
- Yes, for Non-COVID-19 patients
- Yes, for some other purpose (please specify) ________________________________________________

Q17. Has your any of your CRU Staff been reassigned as a result of COVID-19? (check all that apply)

- No
- Yes, voluntary reassignment
- Yes, mandatory reassignment

Q18. Which are being reassigned? (check all that apply)

- Nurses
- Medical assistants
- Nurse assistants
- Lab staff
- Respiratory therapist
- Clerks/secretary
- Dietician
- Other (please specify) ________________________________________________

Q19. Do you currently have any CRU staff in quarantine due to COVID-19 exposure?

- Yes
- No

Q20. About what percent of your staff is impacted by quarantine?

________________________________________________________________

Q21. Compared to normal operations, how has the time spent on these non-clinical activities changed during the COVID-19 pandemic? ("Much Less than Normal" to "Much More than Normal")

|  | Much Less than Normal | Somewhat Less than Normal | About the Same as Normal | Somewhat More than Normal | Much More than Normal |
| --- | --- | --- | --- | --- | --- |
| Protocol reviews |  |  |  |  |  |
| Protocol training |  |  |  |  |  |
| Development and revisions of SOP |  |  |  |  |  |
| Education/professional development |  |  |  |  |  |
| Organization of labs/units |  |  |  |  |  |
| Other (1) |  |  |  |  |  |
| Other (2) |  |  |  |  |  |
| Other (3) |  |  |  |  |  |

Q22. Are you maintaining your regular communications with your research teams?

- Yes
- No

Q23. How frequently have you been providing regular communications with your research teams?

- Daily
- Weekly
- Every two weeks
- Monthly
- Other e.g. "as needed" (please specify) ________________________________________________

Q24. What format(s) are these communications? (check all that apply)

- Email
- Phone
- Video Conference (e.g. Zoom)
- Newsletter
- Other (please specify) ________________________________________________

Q25. Has your CRU staff began meeting to develop a plan and/or timeline for recovery (i.e. returning to normal operations)?

- Yes, we are already meeting
- No, and we have not planned an initial meeting
- No, but we have planned an initial meeting

Q26. Please describe your recovery plan including the timeline briefly:

________________________________________________________________

________________________________________________________________

________________________________________________________________

________________________________________________________________

________________________________________________________________

Q27. Alternatively, you can choose to upload a copy of your plan if you prefer.

Q28. Please provide any additional feedback about your experiences.

________________________________________________________________

________________________________________________________________

________________________________________________________________

________________________________________________________________

**eFigure 2. Impact of Covid-19 On CRUs – RUN Members Interview Guide**

**Questions:**

Your first name, institution, and your role in the institution.

1. To what extent has your CRU returned to “normal” or “near normal” operations?
   - When did this happen?
   - Was it a phased process or “all at once”?
   - Is your CRU’s operations similar to your institution’s other outpatient/inpatient units? If not, what are the differences?
2. How did you decide on reopening your CRU?
   - How was the decision made (e.g., top-down approach from the university, hospital, or state leadership)?
   - Formal and informal meetings? If yes, who were the participants? What are their roles?
   - How are you restarting studies in your unit?
     1. How are you prioritizing them?
   - Who is making those decisions?
     1. E.g. Study team, institution/hospital, CRU independently
   - What, if any, type of data collection instruments or systems are you using to help with reopening?
     1. E.g. Surveys, datasets, etc. Are you willing to share it (them) with others?
     2. Are their types of data collection instruments or systems that RUN could create that would help you and others in your work?
3. What, if any, educational or informational materials have you developed for your different stakeholder groups? (e.g. patients, study teams)
   - Are you willing to share those materials with others in the network?
4. How have your Standard Operating Procedures changed?
5. What is your institution interpretation of ‘Essential Research’ policy discussed in earlier survey?
   - How essential research is further defined during this reopening?
   - Is the PI or OVPR provide CRUs further guidance on essential research?
6. What are the safety measures you are using or considering in your unit now?
   - Please provide specific example (e.g., follow-up swab in a portable unit is being used for study A).
   - Has someone from hospital epidemiology or similar unit inspected your clinic space and provided safety measures feedback?
7. What challenges are you encountering during reopening?
   - Lack of public trust
   - Enrollment pushback
   - Backlog issues
   - Extended clinic hours and weekends?
   - Acquiring, using, and conserving PPE
   - Visitor restrictions
   - Scheduling requests
   - Maintaining physical distance guidelines
   - Workforce available vs # of visit requests
   - **How are you addressing these challenges? – MUST ASK!**
8. Which, if any, COVID-related studies are you currently working on at your unit?
   - HERO-HCQ
   - Convalescent plasma
   - Remdesivir
   - Regeneron outpatient eSPIKE (cases & households)
   - Regeneron inpatient (eSPIKE) (cases)
   - Vaccines studies
9. What are the challenges and solutions for RUN members to consider with these studies?
10. What is your CRU’s takeaway/lessons learned from this pandemic so far?
11. How have you been preparing for future surge?
